# Pandemic preparedness and responses: WHO to turn to in a crisis?

**DOI:** 10.1371/journal.pmed.1003167

**Published:** 2020-05-29

**Authors:** The PLOS Medicine Editors

**Affiliations:** Public Library of Science, San Francisco, California, United States of America and Cambridge, United Kingdom

## Abstract

The PLOS Medicine editors discuss the role of the World Health Organization in pandemic responses.

In the thick of a global pandemic, it should be straightforward to appreciate the role and responsibilities of the World Health Organization (WHO). With a newly emerged coronavirus, SARS-CoV-2, exerting an appalling global toll in terms of lives lost, ill-health, and societal and economic disruption, the organization is a fulcrum on which all efforts to combat the COVID-19 outbreak and manage its consequences must be based. As of April 30, in excess of 3.2 million cases had been recorded, with more than 227,000 deaths attributed to the disease [1]. WHO Director-General Tedros Adhanom Ghebreyesus, who has led the agency since 2017, has been prominent in the response to the coronavirus outbreak, not least in his authoritative public appearances. Yet the rapidity of the pandemic’s growth, and the diverse and apparently tentative responses in certain countries, have created concerns in some quarters about the agency’s capabilities to advise on and respond to disease outbreaks. Indeed, on April 14, a short suspension of US funding for WHO was announced, prompted by alleged suppression of information about the COVID-19 outbreak during its early stages in China [2]. Little is certain about the course and possible conclusion of the current outbreak, save that the actions and attributes of WHO, its structures, and its people will be scrutinized in the minutest possible detail.

WHO is tasked with forcefully representing international resolutions, creating confidence in its unparalleled technical capacity, and acting fairly and responsibly to promote health and wellbeing in all countries, as far as is possible. These countries vary enormously in population size and structure, wealth, political objectives and many other characteristics, of course. Consequently, the element of WHO’s 1946 Constitution that posits achievement of “the enjoyment of the highest attainable state of health … [by] every human being without distinction” may—perversely given its universal appeal—be subordinated to other, more short-term, factors [3]. It is easy to imagine situations in which attaching blame to an international body might be an attractive route to political or economic advantage. WHO’s revenue was about $2.9 billion in 2018 [4], made up of assessed and voluntary contributions, the latter category often linked to specific programmes or aims by donors. Large donors and countries are therefore likely to have, or be perceived to have, influence over its actions and announcements. WHO’s physical and human footprint is also criticized from time to time, and even ardent supporters would concede that, were the agency to be designed and launched today, its Swiss headquarters, along with 6 regional fiefdoms and 150 country offices, could well be reimagined in a much more streamlined fashion.

As we discussed in an Editorial in 2016 [5], WHO’s record in previous infectious disease outbreaks has not always met with unqualified approval. The agency’s response to the 2012–2014 Ebola outbreak in West Africa, under previous Director-General Margaret Chan, was seen to have been plagued by delay and dysfunction. In the subsequent reports that investigated WHO’s perceived failings, it was noted that the organization had, for example, previously cut a substantial proportion of its emergency response capability, and “lacked the governance needed to coordinate multiple stakeholders” in the response to a disease outbreak [6]. Essentially, there was a sense that the organization had been trying to do too many things with too few resources, and making questionable strategic decisions in the process.

The recent suspension of US funding for WHO has elicited criticism from many in the health arena, including PLOS [7]. Additional political manoeuvring has followed [8], and subsequently China has trumped the announcements by pledging an additional $30 million in funding, noting that WHO had been “actively fulfilling its duties and upholding an objective, scientific and impartial stance” on the disease outbreak [9]. These opportunistic political gambits could well continue in longer campaigns seeking to acquire plaudits for perceived (but at this stage perhaps ephemeral) successes in addressing the continuing outbreak, alongside creative attribution of responsibility for early, and possibly onging, errors and omissions in country-specific pandemic responses; wilful misinformation must also be considered as a factor.

We contacted a number of commentators for their views on the emerging debate around WHO’s role in the current outbreak, and Margaret Kruk, of the Harvard T.H. Chan School of Public Health, argues that “WHO plays an indispensable role in our shared health and it is one of the few institutions that is seen as credible in countries at a time that health and science are increasingly politicized. But it is hamstrung by insufficient, strings-tied funding and a governance structure that precludes its ability to speak uncomfortable truths for fear of offending member countries. The goal of reforms should be to build a technically stronger, better funded, and more independent WHO”. Although far too early to make definitive judgments about individual country or agency actions during the current pandemic, we can anticipate a frank debate about the capabilities and actions of WHO throughout this extraordinary time. Among thoughts that come to mind are, first, that political involvement with or by WHO is regrettable, with its parent organization, the United Nations, the forum for this purpose. Second, the experiences of the current pandemic need to be put to good use to prepare WHO and countries for future disease outbreaks—how do the agency’s capabilities and infrastructure, and indeed those of country public health bodies, need to be strengthened and adapted to this end? It may be that the function of WHO needs to be refocused on convening expertise and providing normative guidance for health goals, with a distinct entity, akin to UNAIDS, adopting responsibility for outbreak surveillance and responses. Finally, a global agency for health will remain essential, and all governments should seek to work with rather than counter to WHO as an essential partner in promoting the increasingly interconnected state of the world’s health.
